# Supplementary material for: Real-World Impact of Deep Targeted Sequencing on Erythrocytosis and Thrombocytosis Diagnosis: A Reference Centre Experience
Source: Cancers (Basel). 2024 Sep 14;16(18):3149. doi: 10.3390/cancers16183149 (PMC11430442; doi:10.3390/cancers16183149)
Supplement: Supplementary file 1 [file cancers-16-03149-s001.zip › cancers-3188970-supplementary.pdf]

Next generation sequencing (NGS)

NGS had been performed using an Ion Torrent Proton^{TM} sequencer (Life Technologies, Palo Alto, CA). Data analyses had been performed using Ion Reporter 4.4 Software (Life Technologies), which identified SNVs and indels. Samples were sequenced with with an average depth of coverage of over 2,000 reads by amplicon.

Gene panel

Exons and splicing regions of the following genes were sequenced: *ASXL1, BCOR, BCORL1, CALR, CBL, CEBPA, CSF3R, DNMT3A, EGLN1, EPAS1, EPOR, ETV6, EZH2, FLT3, IDH1, IDH2, JAK2, KDM6A, KIT, KMT2A, KRAS, MPL, NF1, NPM1, NRAS, PHF6, PRPF40B, RAD21, RUNX1, SETBP1, SF3A1, SF3B1, SH2B3, SMC1A, SRSF2, STAG2, TET2, THPO, TP53, U2AF1, VHL, WT1, ZRSR2.*

Supplementary Table S1. Description of patients with *JAK2* exon 12 mutation detected by Next Generation Sequencing (NGS)

**Age at NGS (y)**	**Sex**	**Maximum Hb**	**Maximum Hct**	**Leu/plat**	**Serum EPO**	**Prior thrombosis**	**Coding region change**	**Aa change**	**VAF (%)**	**Clinical events**
80	Male	18.3	57.8	Normal	Low	DVT	c.1614_1616delCAAinsATT	p.H538_K539delinsQL	24.89	No
62	Male	18	55.8	Normal	Low	DVT	c.1614_1616delCAAinsATT	p.H538_K539delinsQL	38	SMF
37	Male	19.3	60.7	Normal	Low	No	c.1613_1616delinsT	p.H538_K539delinsL	7.6	No
78	Female	18.1	56.3	Normal	Low	Ischemic stroke	c.1640_1641insGTTTCACAAAATCAGAAATGAAGATTTGATATT	p.I546_F547ins11	15.7	No
81	Male	19	58.7	Normal	Low	No	c.1615_1616delAAinsCT	p.K539L	8.3	No

y: years, Hb: haemoglobin (g/dL), Hct: haematocrit (%), Leu: leucocytes, Plat: platelets, EPO: erythropoietin, Aa: amino acid, DVT: deep venous thrombosis, VAF: variant allele frequency, SMF: secondary myelofibrosis.

Supplementary Table S2. Description of patients with familial erythrocytosis variants detected by Next Generation Sequencing (NGS)

**Age at NGS (y)**	**Sex**	**Family history**	**Maximum Hb**	**Maximum Hct**	**Leu/plat**	**Prior thrombosis**	**Gene**	**Coding región change**	**Aa change**
74	Male	Son	18.3	55.5	Normal	No	*EPOR*	c.1460A>G	p.N487S
51	Male	No	18	55.3	Normal	TIA	*EPAS1*	c.1121T>A	p.F374Y
54	Male	Father and uncle	20.3	60.6	Normal	No	*EPAS1*	c.1591C>T	p.P531S
38	Female	Mother, brother	17.3	54.3	Normal	No	*EGLN1*	c.809G>A	p.G270E
50	Male	Yes*	18	53.4	Normal	No	*EGLN1*	c.809G>A	p.G270E
77	Female	No	18.1	53.9	Normal	No	*EGLN1*	c.809G>A	p.G270E
53	Male	No	18.7	58.9	Normal	No	*EPAS1*	c.1700T>C	p.M567T
73	Female	No	17.7	52.4	Normal	No	*EGLN1*	c.311C>T	p.S104F

Hb: haemoglobin (g/dL), Hct: haematocrit (%), Leu: leucocytes, Plat: platelets, Aa: amino acid, TIA: transient ischemic attack. *Unspecified. Shaded rows show patients with variants of uncertain significance.

Supplementary Table S3. Description of patients with erythrocytosis and clonal hematopoiesis of indeterminate potential (CHIP)

**Age at NGS (y)**	**Sex**	**Max Hb**	**Max Hct**	**EPO**	**Bone marrow**	**Gene**	**VAF (%)**	**Coding region change**	**Aa change**	**Classification**	**Final diagnosis**
69	M	19.1	56.5	High	Not performed	*KRAS*	3.62	c.35G>A	p.G12D	Pathogenic	SAS
76	M	18.3	54	High	Not performed	*DNMT3A*<br>*SRSF2*	13.82<br>4.76	c.2245C>T<br>c.284C>A	p.R749C<br>p.P95H	Pathogenic<br>Pahogenic	Idiopathic
72	F	16.8	51.2	Normal	Not performed	*TET2*	3	c.935delA	p.N312fs	Likely pathogenic	Idiopathic
58	M	18.5	54.9	Normal	Not performed	*DNMT3A*	3.37	c.1040T>G	p.L347R	VUS	Idiopathic
17	M	18	53.6	Normal	Not performed	*CEBPA*	14	c.589_590insA CCCGC	p.P196_P1 97ins	Pathogenic	Metabolic syndrome
73	M	17.9	55.6	Unavailable	Not performed	*DNMT3A*	3.06	c.1792C>T	p.R598*	VUS	Idiopathic
66	M	19.3	57	Normal	Not performed	*TET2*	9.88	c.2656C>T	p.Q886*	Pathogenic	Idiopathic
52	M	19.1	57.7	Normal	Not performed	*DNMT3A*	7.8	c.1135C>T	p.R379C	VUS	Smoking
57	M	19.5	58.9	Normal	Inconclusive	*RUNX1*	3.14	c.1244A>G	p.Q415R	VUS	Idiopathic

M: Male, F: Female, Max Hb: maximum haemoglobin (g/dL), Max Hct: haematocrit (%), Leu: leucocytes, Plat: platelets, VAF: variant allele frequency, Aa: amino acid, VUS: variant of uncertain significance, SAS: sleep apnoea syndrome.

Supplementary Table S4. Description of patients with variants that led to diagnosis of essential thrombocythemia (ET) or familial thrombocytosis

**Age at NGS (y)**	**Sex**	**Maximum plat**	**Hb**	**Leu**	**Prior thrombosis**	**Bone marrow histology***	**Gene**	**VAF**	**Coding region change**	**Aa change**
71	Female	634	13.7	7.2	DVT	Consistent	*TET2*	4.98%	c.5152G>T	p.V1718L
62	Male	1,001	14.5	9.6	No	Consistent	*JAK2*	24.1%	c.2618A>C	p.D873A
59	Male	585	14.2	6.6	No	Consistent	*SF3B1*	30.8%	c.2098A>G	p.K700E
44	Female	1,000	13.4	8.2	No	Consistent	*JAK2*	47.68%	c.1541C>G	p.T514R
37	Male	1,020	13.9	7.4	No	Consistent	*JAK2*	50,1%	c.2047A>G	p.R683G
74	Male	543	14.4	8.3	No	Consistent	*TET2*	3.5%	c.1960C>T	p.Q654*
21	Female	828	13.8	6.6	No	Inconclusive	*THPO*	47.15%	c.25G>A	p.V9M
81	Female	826	12.2	16.9	No	Inconclusive	*IDH2*	3.03 %	c.419G>A	p.R140Q
76	Female	691	14.1	5.1	DVT	Inconclusive	*ASXL1*<br>*TET2*	48.8%<br>29.3%	c.2898_2900delAGG<br>c.3998_4012delTGGCACCAACATATA	p.G966del<br>p.M1333_Y1337del
59	Female	827	14	6.9	No	Inconclusive	*DNMT3A*	4.61%	c.2322G>C	p.E774D
44	Female	555	14.2	9.5	No	Inconclusive	*CALR*	48.51%	c.1153_1155delAAG	p.K385del
58	Female	830	13.5	6.8	No	Normal	*ASXL1*	46%	c.4072C>G	p.P1358A
49	Female	800	14.3	10	No	Normal	*TP53*	2.75%	c.659A>G	p.Y220C
55	Female	525	12.6	12	No	Normal	*FLT3*	47.04%	c.2822C>T	p.S941L
66	Female	657	13.6	8	No	Inconclusive	*THPO*	45,4%	c.957G>C	p.Q319H

Hb: haemoglobin (g/dL), Leu: leucocytes (x10^9/L), Plat: platelets (x10^9/L), Aa: amino acid. DVT: deep venous thrombosis. Shaded rows show cases with a familial disorder. *Bone marrow histology classified as consistent or inconclusive for ET diagnosis or normal.
